# 

**Published:** 1912-03

**Authors:** 


					MARCH, 1912.
Vol. XXX. No. 115.
THE
BRISTOL
MEDICO-CHIRURGICAL
THE BRISTOL MED1C0-CH1RURG1CA
Editor: R. SHINGLETON SMI
WITH WHOM ARK ASSOCIATE
J. MICHELL CLARKE, M.A.,
J. LACY FIRTH, M.S., M.D., JAMES !
P. WATSON-WILLIAMS, M.D., Assistant
Editorial Sicritary: J. A. NIXON, B.A., M.B.
BRISTOL: J. W. ARROWSMITH LTD.
London : J. k A. Churchill, 7 Great Marlborough Street.
Price One Shilling and Sixpence.

				

